# Recycled Brewer’s Spent Grain (BSG) and Grape Juice: A New Tool for Non-Alcoholic (NAB) or Low-Alcoholic (LAB) Craft Beer Using Non-Conventional Yeasts

**DOI:** 10.3390/foods13040505

**Published:** 2024-02-06

**Authors:** Laura Canonico, Alice Agarbati, Francesca Comitini, Maurizio Ciani

**Affiliations:** Department of Life and Environmental Sciences, Polytechnic University of Marche, Via Brecce Bianche, 60131 Ancona, Italy; l.canonico@univpm.it (L.C.); a.agarbati@univpm.it (A.A.); f.comitini@univpm.it (F.C.)

**Keywords:** non-alcoholic beer, low-alcoholic beer, Italian grape ale, non-*Saccharomyces*, brewer’s spent grain

## Abstract

Non-alcoholic beer (NAB) and low-alcoholic beer (LAB) are taking over the market with growing sales. Sustainable recycling and valorization of exhausted brewer’s spent grain (BSG) coming from craft beer is a relevant issue in the brewing process. In this work, recycled BSG and BSG + GJ (supplemented with 10% grape juice) were used as a wort substrate to inoculate *Lachancea thermotolerans*, *Wickeramhomyces anomalus*, *Torulaspora delbruecki* and *Pichia kluyveri* non-conventional yeasts to produce NABLAB craft beer. Results showed that wort composed of only recycled BSG produced appreciated NAB beers (ethanol concentration from 0.12% to 0.54% *v*/*v*), while the addition of 10% grape juice produced LAB beers (ethanol concentration from 0.82 to 1.66% *v*/*v*). As expected, volatile compound production was highest with the addition of grape juice. *L. thermotolerans* showed lactic acid production, characterizing both worts with the production of ethyl butyrate and isoamyl acetate. *T. delbrueckii* exhibited relevant amounts of hexanol, phenyl ethyl acetate and β-phenyl ethanol (BSG + GJ). *W. anomalus* and *P. kluyveri* showed consistent volatile production, but only in BSG + GJ where fermentation activity was exhibited. The overall results indicated that reused BSGs, non-conventional yeasts and grape juice are suitable bioprocesses for specialty NABLAB beer.

## 1. Introduction

The popularity of craft and specialty beers, together with a healthier society, have defined new trends in the brewing industry [1]. New flavors and styles, but also functional and non-alcoholic beers, are the main drivers of product diversification. Therefore, fermentation management using new starter yeasts represents an opportunity where the brewer can easily diversify the product to satisfy consumer desires [2,3,4,5]. In this context, brewers interested in meeting consumers’ expectations of more healthy products and in expanding their assortment of beers must increase their production of non-alcoholic beers (NAB) or low-alcoholic beers (LAB) that are taking over the market. It has been reported that average sales in Europe have increased by 50% over the last 15 years [6]. Depending on the countries’ laws and the producers’ equipment, NAB can contain 0–0.5% ethanol [7]. In the EU, no alcoholic beverages containing more than 1.2% ABV are allowed to bear health claims, while nutrition claims should refer only to low alcohol levels or to the reduction of alcohol content or energy content [8]. In the production of these specialty beers, the reduction of ethanol content is performed during fermentation by biological methods (modifying yeast fermentation or strains) or physical methods applied after fermentation (thermal or membrane processes) [2]. The use of biological methods, based on the conscious use of selected non-conventional yeasts, different from *Saccharomyces* traditionally inoculated as starter cultures for brewing, could be a useful approach to produce NAB LAB, as non-*Saccharomyces* yeasts have a limited ability to ferment wort sugars [3,9,10,11,12,13,14,15]. Several non-*Saccharomyces* yeasts have been investigated to produce non-alcoholic beer with promising results, e.g., *Saccharomycodes ludwigii*, *Zygosaccharomyces rouxii* [12], *Cyberlindnera* spp. [3,16,17] *Hanseniaspora* spp. [13], *Torulaspora delbrueckii* [13,15,18,19,20,21,22] and *Pichia kluyveri*, *Kluyveromyces lactis* [21,23].

Furthermore, these yeasts possess metabolic activities that differ from *Saccharomyces* and may confer peculiar aromatic traits for the natural production of NAB LAB. However, one very crucial aspect for the use of microorganisms in food applications is the regulatory concern regarding the safety of microbial cultures, while *Saccharomyces cerevisiae* possesses an extensive and well-documented history of safe use [24].

Brewer’s spent grain (BSG) is the main by-product of a brewery, corresponding to almost 85% of the total residues generated from raw materials (usually from malted barley, wheat, rye, oats or other grains used in the brewing process) [25,26]. BSG has a broad spectrum of possible uses. Traditionally, spent grain has been used for animal feed and constitutes a valuable source of compounds that can be used as functional bioactive ingredients in human nutrition with numerous benefits for human health [27] or as compost and energy production that can contribute to a more sustainable circular industry. On the other hand, exhausted BSG coming from craft beer production possesses quite relevant sugars and other valuable compounds that could be further used. Recently, Canonico and co-workers [28] investigated different non-conventional yeast strains to produce NAB LAB beers using recycled and exhausted BSG. Wort from the recycled BSG inoculated with selected non-conventional yeasts was a favorable combination for producing NAB LAB beers.

In this study, a wort obtained from exhausted BSG coming from the mashing of a Pils wort was evaluated with the addition of 10% vol. Verdicchio grape juice as a fermentation substrate to evaluate the use of selected non-conventional yeast species. The aim was to valorize and reuse a by-product of craft beer to produce a NAB LAB Italian grape ale-style beer.

## 2. Materials and Methods

### 2.1. Yeasts Strains

The non-conventional yeast strains, coming from the Department of Life and Environmental Sciences (DiSVA) Collection, and the *S. cerevisiae* US-05 (Fermentis, Lesaffre, France) starter strain used as control are reported in Table 1.

The five yeasts strains were selected taking into account the results of a previous work regarding the analytical and sensory profile of beers [28].The yeast strains used in the trials were cultivated and maintained in YPD agar medium (10 g/L yeast extract, 20 g/L peptone, 20 g/L glucose and 18 g/L agar) at 4 °C for short-term storage, while for long-term storage, YPD broth supplemented with 40% (*w/v*) glycerol at −80 °C was used.

### 2.2. Brewer’s Spent Grain (BSG) and Substrate of Fermentation/Recycled BSG to Produce Worts with Low Sugar Content: BSG and BSG + GJ (10% Grape Juice Added)

A PILS wort (170 kg of malt Pils in water at 35 °C) was used to obtain BSG used for the preparation of a wort with low sugar content.

The PILS wort (lager beer, Pilsner style) was used to prepare 1000 L of Pils craft beer at the Birra dell’ Eremo craft brewery (Assisi, Italy) after the addition of Cascade hops during the boiling phase. The main wort (1000 L) had the following composition: pH 5.5, density 12.3 °P and 20 IBU. After the filtration step at 78 °C, the wort (after boiling and the addition of Cascade hops and the whirlpool and cooling phases) was inoculated for the industrial fermentation process. After this, the exhausted BSG was further filtered using water at 78 °C and four hundred liters was collected and uniformed. The wort from the exhausted BSG had the following composition: pH 5.5; density 1.2 °P; glucose (0.80 g/L); sucrose (0.11 g/L); maltose (3.90 g/L); yeast assimilable nitrogen (YAN) (4.47 mg N/L); polyphenols (28.33 mg/L). A total of 15 L of combined wort from exhausted BSG was added with 18 g of Cascade hops and boiled for 1 h. The hopped substrate was divided equally into two batches. In the first batch, it was kept as is and named recycled BSG (BSG), while, in the second, 10% of pasteurized Verdicchio grape juice was added (vintage 2017 (BSG + GJ wort)). The main analytical parameters of the Verdicchio grape juice used were initial sugar (glucose and fructose) (212 g/L); pH (3.22); total acidity (4.58 g/L); malic acid (2.5 g/L); total SO_2_ (27 mg/L); YAN 60 mgN/L). The BSG + GJ wort showed the following initial composition: glucose (16.7 g/L); fructose (15.1 g/L); sucrose (0.46 g/L); maltose (3.91 g/L); YAN (53.91 mgN/L); polyphenols (75.83 mg/L). The two worts obtained were used separately as substrates for setting up micro-fermentations.

### 2.3. Micro-Fermentations

The fermentation trials were carried out in triplicate at 20 °C ± 2 °C in 500 mL flasks with hydraulic valves containing 450 mL of both worts (BSG and BSG + GJ). The pre-cultures were grown for 48 h in 10% malt extract at 20 °C ± 2 °C. After incubation, the cells were collected by centrifugation (2000× *g* for 5 min), resuspended in sterile water and inoculated at 1 × 10^6^ cells/mL (estimated by a Thoma–Zeiss chamber) in the flasks containing both worts (BSG and BSG + GJ) and incubated at 20 °C ± 2 °C. To evaluate the fermentation evolution, the weight loss due to the CO_2_ evolution was monitored and recorded daily until reaching a constant value. After fermentation and a week at 4 °C, 1.5 g/L of sucrose was added to the beers after evaluating the residual viable yeasts, and the beers were bottled. After the addition of sugar, the bottles were kept at 18–20 °C for about 7–10 days for the bottle refermentation phase, and then stored at 4 °C. 

### 2.4. Microbiological and Chemical Analysis

The evaluation of viable cell counts was conducted at the start of the fermentation process. A DA-300 specific gravity meter (Kyoto Electronics Manufacturing, Kyoto, Japan) was used to measure specific gravity. All these measurements were converted to densities and then to degrees Plato, following the indications of Brown and Hammond [29]. The enzymatic method (Megazyme, Wicklow, Ireland) was used to determine glucose, sucrose and maltose. The free amino nitrogen was evaluated using the procedures described by Dukes and Butzke [30]. The volatile acidity and pH were performed according to the Official European Union Methods EC 2000. Ethanol was evaluated by gas–liquid chromatographic analysis [31].

The concentration of the volatile compounds was detected using the solid-phase microextraction (HS-SPME) method. An amount of 5 mL of beer was placed in a vial containing 1 g NaCl closed with a septum-type cap. The extraction was carried out with magnetic stirring for 10 min at 25 °C. After that, the sample was heated to 40 °C after the addition of the internal standard (3-octanol) (Sigma-Aldrich, St. Louis, MO, USA) at a concentration of 1.6 mg/L. Divinylbenzene/carboxen/polydimethylsiloxane (DVB/CAR/PDMS) fiber (Sigma-Aldrich) was inserted into the vial headspace for 30 min. The fiber was desorbed by insertion into a GC-2014 (Shimadzu, Kjoto, Japan) injector for 5 min. A Supelcowax 10 (length, 60 m; internal diameter, 0.32 mm) glass capillary column was used. The fiber was inserted in the split–splitless mode, as reported by Canonico et al. [31]. The compounds were identified and quantified by comparisons, with calibration curves for each compound. Specific enzymatic kits (Megazyme, Wicklow, Ireland) were used to determine the concentrations of glucose sucrose, maltose and lactic acid (kit k-masug, Megazyme, Wicklow, Ireland) according to the manufacturer’s instructions. The analyses were conducted after the primary fermentation.

### 2.5. Statistical Analyses

The statistical analyses of the main analytical characters and volatile compounds of the beers were evaluated by analysis of variance (ANOVA). Duncan’s test was used to determine the significant differences. The results were considered significant if associated with a value <0.05. Volatile compounds were also evaluated by using principal component analysis (PCA) to discriminate between the means and the variability due to the inoculated strains and different worts (BSG and BSG + GJ). Statistical software package JMP 11^®^ (SAS, Cary, NC, USA) was used for statistical analysis.

## 3. Results

### 3.1. Fermentation Kinetics

**Figure 1:**
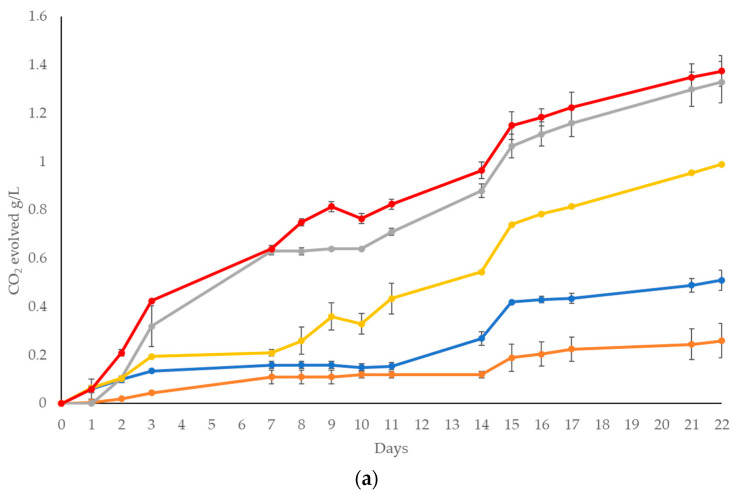

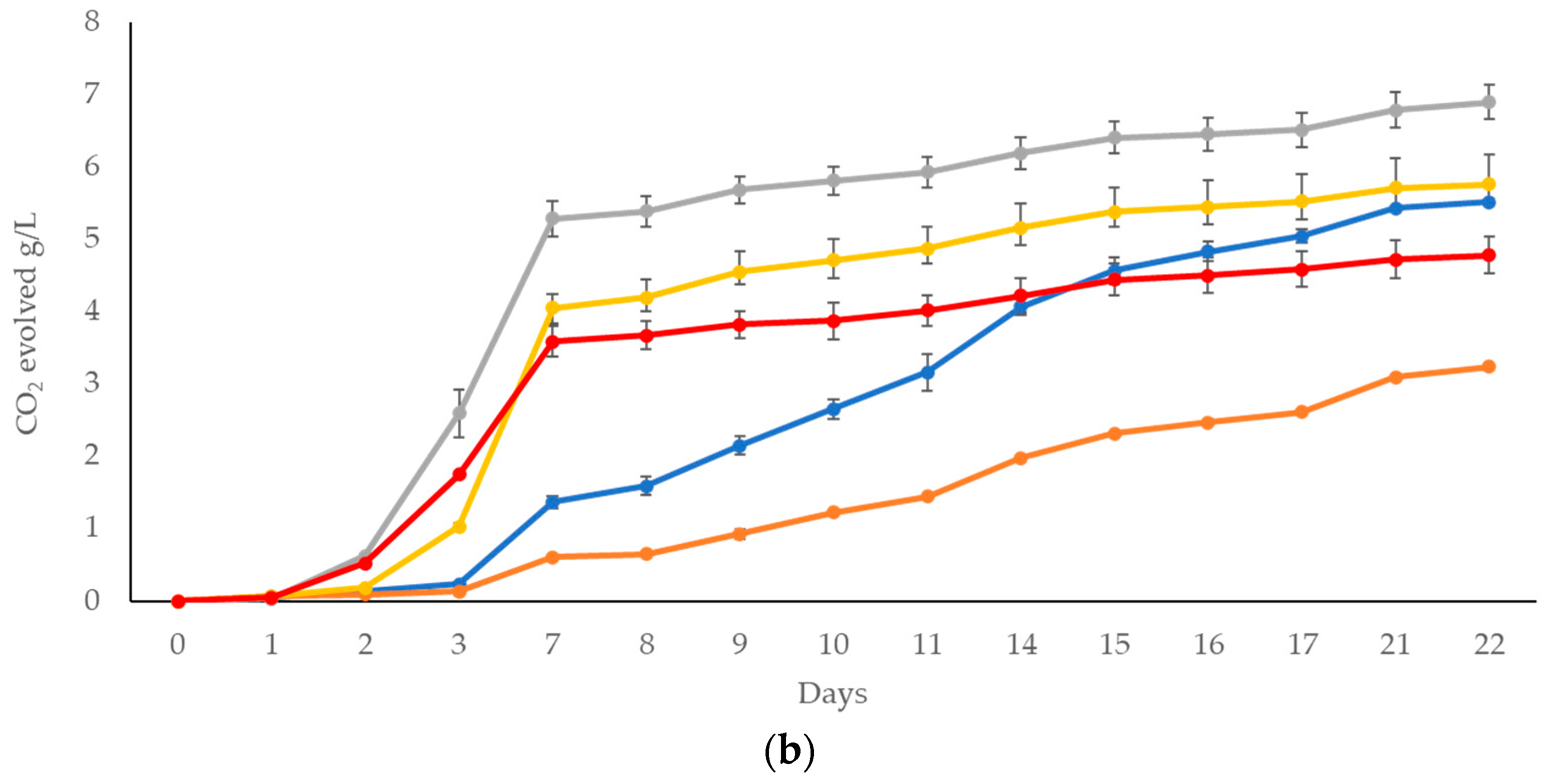
Fermentation kinetics of *W. anomalus* DiSVA 2 (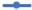 ); *P. kluyveri* Disva 1076 (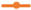); *T. delbrueckii* DiSVA 254 (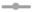); *L. thermotolerans* DiSVA 322 (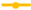); and *S. cerevisiae* (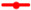). (**a**) Wort from BSG; (**b**) wort from BSG + GJ.

The data of the fermentation evolution are reported in Figure 1. The CO_2_ evolution in the trials using wort from BSG (Figure 1a) showed relevant differences among the strains tested. *T. delbrueckii* DiSVA 254 exhibited a similar trend to the *S. cerevisiae* starter strain US05, with a total evolved CO_2_ of 1.33 g/450 mL, while L. *thermotolerans* showed an intermediate fermentation kinetics compared with the strains tested. *W. anomalus* and *P. Kluyveri* exhibited the lowest fermentation performance in comparison with the other strains.

As expected, in the trials carried out in the BSG + GJ wort (Figure 1b), an increase in evolved CO_2_ was shown. The fermentation trend of the different strains tested followed the one reported in Figure 1a. The only exception was *S. cerevisiae* commercial strain US05, which exhibited a rapid first part of fermentation and then slowed down with a final evolved CO_2_ (4.78 g) slightly lower than *T. delbrueckii* and *L. thermotolerans*. *W. anomalus* and *P. kluyveri* showed the slowest fermentation trend and final evolved CO_2_ in comparison with the other fermentation trials.

### 3.2. The Main Analytical Characteristics of Final Products

The results regarding the main analytical characteristics of beers coming from BSG and BsG + GJ worts are reported in Table 2.

All strains consumed all sucrose and glucose, with the exception of *P. kluyveri* (0.11 g/L of glucose). The ability to metabolize maltose, however, was variable among the species, with a significantly higher residue of this sugar in the fermentations carried out by *W. anomalus* and *P. kluyveri*. This result could be related to their low fermentation ability, as shown in Figure 1a. Regarding the values of YAN, the data showed a limited reduction in all fermentations tested, indicating a low general yeast consumption (initial concentration = 41.47 mg/L). The evaluation of lactic acid produced during fermentations showed the ability of *L. thermotolerans* to produce lactic acid during sugar fermentation, while other species tested showed lower values comparable to the initial amount present in the wort. The results of the ethanol content were consistent with the fermentation activity. *S. cerevisiae* showed the highest ethanol production (0.54% *v*/*v*). *T. delbrueckii* and *L. thermotolerans* showed very similar final ethanol concentrations, while the lowest amounts were found in the *P. kluyveri* trials (0.12% *v*/*v*). All the fermentation trials could be considered NAB beers as they showed ethanol concentrations equal to or less than 0.5%. When analyzing the data relating to the polyphenolic content, a significantly higher content was found in the fermentation trials carried out in the *W. anomalus* and *S. cerevisiae* strains.

The results of the main analytical characteristics of beer samples with the addition of 10% grape juice are reported in Table 3.

Also, in this case, all the species analyzed were able to metabolize the glucose (fructose) and sucrose present in the wort, except for *P. kluyveri*, which showed glucose and sucrose residues in the final products. The data relating to maltose consumption showed greater variability, e.g., a significantly higher residue was shown in *S. cerevisiae* and *W. anomalus*; even in this case, a decrease in YAN content was observed. In the fermentation carried out with *P. kluyveri*, a significant quantity of YAN was found, indicating a reduced metabolic activity, while a significantly higher consumption of YAN was exhibited in the *T. delbrueckii* trials. The quantity of polyphenols found was lower than that of the initial substrate. The greatest reduction was found in the *T. delbrueckii* trials, where the polyphenolic components were almost completely removed, while the lowest reduction of the polyphenolic compounds was found in *P. kluyveri*.

The fermentations conducted using this substrate showed the ability of *L. thermotolerans* to produce lactic acid, which exhibited a significant amount of this compound. Finally, the ethanol production, in line with the fermentation trend, varied from 1.66 (% *v*/*v*) *T. delbrueckii* to 0.82 (% *v*/*v*) *P. kluyveri*, which could be considered LAB beers.

### 3.3. Volatile Compounds

The main volatile compounds formed in beers coming from exhausted BSG wort are reported in Table 4. 

The trials conducted with *L. thermotolerans* and *S. cerevisiae* produced the highest and most relevant amounts of ethyl butyrate (pineapple flavor) and isoamyl acetate (banana aroma). Moreover, *S. cerevisiae* led to a significant amount of diethyl succinate and geraniol, while *T. delbrueckii* showed a significant increase in hexanol (under the OAV value). Linalool, phenyl ethyl acetate and nerol did not show significant differences among the strains tested.

The results of the volatile compounds detected in the wort with the addition of 10% Verdicchio grape juice are reported in Table 5.

As expected, in BSG wort, a general increase in volatile compounds was shown. *L thermotolerans* and *S. cerevisiae* confirmed the highest production of isoamyl acetate (banana flavor) exhibited in the BSG trials. The highest quantity of ethyl butyrate (pineapple flavor) was obtained in the trial carried out with *L. thermotolerans*, followed by *S. cerevisiae* US05 (0.54 mg/L) and *T. delbrueckii* (0.46 mg/L). Ethyl hexanoate was produced in significant quantities by *S. cerevisiae* (0.34 mg/L), followed by *T. delbrueckii* (0.22 mg/L) and L. *thermotolerans* (0.15 mg/L) (OAV = 0.17–0.20). Hexanol was found only in the trials fermented by *T. delbrueckii* and *L. thermotolerans*.

The monoterpenes linalool and geraniol were recorded in all the tests in negligible quantities, while the concentrations of linalool (lavender and hop aroma) varied between 0.01 mg/L and 0.04 mg/L above the OAV, while geraniol (floral aroma) presented concentrations ranging from 0.01 mg/L to 0.10 mg/L. As regards phenylethyl acetate (fruity and honey aroma), the highest quantity was detected in the *T. delbrueckii* trials. Finally, the content of β-phenyl ethanol (rose aroma) was found to be the highest in the tests conducted with *T. delbrueckii* and *W. anomalus*.

**Figure 2:**
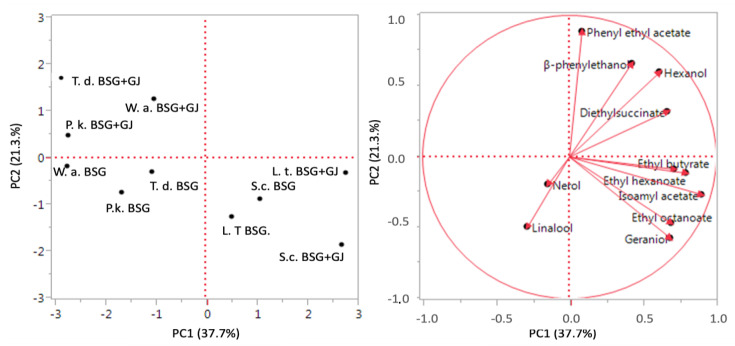
Principal component analysis (PCA) applied on the main volatile compounds in beer coming from different yeast strains and wort (BSG and BSG + GJ) fermentation. The variance explained by PCA is PC 1 37.7% *x*-axis and PC 2 21.3% *y*-axis.

The main volatile compounds coming from the two sets of fermentations with two different substrates (BSG and BSG + GJ) were used in principal component analysis (PCA) (Figure 2). The total variance explained was 59% (PC1 37.7% and PC2 21.3%). As expected, a relevant difference was determined by the addition of 10% vol. grape juice. The trials carried out with the same strain but in different substrates (with and without the addition of Verdicchio grape juice) were placed separately into two distinct quadrants (up and lower left quadrant, respectively). However, *L. thermotolerans* and *S. cerevisiae* were grouped in the same lower right quadrant, indicating their influence on volatile compound production in both substrates.

## 4. Discussion

In recent years, in the craft beer sector, there has been a growing interest in producing healthy products, with a fast development of NABLAB, and in the valorization of by-products coming from beer production, especially BSG [4,6,32,33]. In this work, the reuse of BSG as a fermentation substrate for both the environmental and economic sustainability of the beer production process is considered since little is known about the use of by-products and waste generated by craft breweries [33]. In the craft beer process, BSG still contains significant amounts of sugars and other compounds that could be further used, so different modalities to enhance the sustainability need to be further investigated [34] in order to develop new processes [35]. 

As reported by Canonico et al. [28], exhausted BSG may be a suitable substrate for brewing new NABLABs using non-conventional yeasts. On the other hand, the possibility of obtaining a beer with the addition of natural ingredients with a distinctive aromatic note but with low-alcohol content could be investigated and could be a valuable approach to obtain a specialist beer style.

In this regard, the wort from BSG was added with 10% Verdicchio grape juice using selected non-conventional yeasts. The Verdicchio grape juice addition could be a strategy to obtain a pleasant product but with a low-alcohol content. In the craft beer sector, the addition of natural ingredients is a suitable way to differentiate the wort, producing different beer styles. The communion between beer and wine, called Italian grape ale (IGA), is defined by BJCP (Beer Judge Certification Program, 2015). This new style of beer is characterized by using raw materials linked to wine [31,32,33,34]. Generally, these beers are distinguished by high ethanol content, low bitterness and low pH [36]. Here, the possibility is proposed to obtain an IGA with a low-alcohol content by the reuse of BSG in craft beer production. Indeed, different studies have analyzed IGA related to different grape use, fermentation processes and sensorial aspects [32,33], but there are no studies regarding the production of low-alcohol grape ale with non-conventional yeasts valorizing BSG used as the substrate. All strains tested here led to IGA with low-alcohol content but with fruity and flowery notes. This result would allow the brewer to choose based on the type of product or strain that best lent itself to the final beer. Furthermore, this fermentation behavior can be proposed for functions of different types of grape for IGA. 

Here, the fermentation without the use of grape juice led to NAB beer, confirming the results obtained in a previous work [28]. All the strains tested here showed low ethanol production (from 0.12% to 0.54% *v*/*v*), thus exploitable for NAB beer, while, with the addition of 10% grape juice, they exhibited an ethanol content more suitable for LAB beer (from 0.82 to 1.66% *v*/*v*). As expected, the overall amounts of volatile compounds were in the highest amounts with the addition of grape juice. Ethyl butyrate (pineapple flavor) and isoamyl acetate (banana aroma) were the most relevant volatile compounds, as previously found [11,23]. The lactic acid production, together with the favorable volatile compounds exhibited by *L. thermotolerans*, could be useful in sour beer production. Indeed, this species, together with *S. cerevisiae*, produced (in both worts) significant amounts of ethyl butyrate and isoamyl acetate. This result was confirmed by PCA analysis, showing the positive effect of the grape juice addition on volatile compound production and distinguishing *L. thermotolerans* and *S. cerevisiae* from the other species. *T. delbrueckii* produced relevant amounts of ethyl hexanoate, phenylethyl acetate and β-phenyl ethanol (in wort added with grape juice). *W. anomalus* and *P. kluyveri* species showed consistent volatile production, but only in wort with grape juice, where a fermentation activity was exhibited. Significant results were related to the OAVs, since the mentioned volatile compounds were very close to or above the OAVs. These results, and the preliminary impressions on the beers tasted (without defect), are in agreement with the analytical and sensory analysis shown in the previous work [28]. 

The overall results showed that exhausted BSGs from craft beer production processes could be a valuable substrate if coupled with selected non-conventional yeasts to produce distinctive NABLAB aromatic beer. Indeed, this brewing process may be a valuable double tool with regard to (i) recycling a by-product and (ii) valorizing it to setup different beer styles combining low ethanol production and valuable volatile profile. For the application of this brewing process, further investigations should be directed to set up the fermentation. An important aspect regarding the functional and probiotic features of yeast strains is that the resulted beverage may be considered a functional drink with the appropriate features. On the other hand, the safety aspect of microorganisms in food applications regulated by the EFSA committee is a relevant feature. Indeed, apart from *W. anomalus*, non-conventional yeasts tested are not on the qualified presumption of safety (QPS) list or are not recommended for QPS status in European countries [37]. For these reasons, further investigations regarding the safety aspects of these yeasts are needed.

## Figures and Tables

**Table 1:** Taxonomic classification, strain code and source of isolation of the yeast strains tested and *S. cerevisiae* commercial strain US-05 (Fermentis, Lesaffre, France).

Species	Strain Code (DiSVA Collection)	Source of Isolation
*Lachancea thermotolerans*	322	Grapes
*Pichia kluyveri*	1076	Grapes
*Wickerhamomyces anomalus*	2	Bakery
*Torulaspora delbrueckii*	254	Papaya leaves
*Saccharomyces cerevisiae*	US05	Commercial starter

**Table 2:** The main analytical characteristics of the final products from BSG wort. Data are the means ± SD. Data with different superscript letters (^a,b,c,d^) within each column (Duncan tests; *p* < 0.05). The initial BSG wort characteristics were as follows: glucose (0.80 g/L); sucrose (0.11 g/L); maltose (3.90 g/L). YAN (41.47 mg/L); polyphenols (28.33 mg/L).

Fermentations Trials	Glucose (g/L)	Sucrose (g/L)	Maltose (g/L)	YAN (mg/L)	Polyphenols (mg/L)	Lactic Acid (g/L)	Ethanol (% *v*/*v*)
*Wickeramhomyces anomalus* Disva 2	ND *	ND	3.53 ± 0.02 ^a^	31.03 ± 0.45 ^a^	23.33 ± 1.18 ^a^	0.17 ± 0.00 ^c^	0.27 ± 0.00 ^c^
*Pichia kluyveri* Disva 1076	0.11 ± 0.04 ^a^	ND	3.54 ± 0.04 ^a^	38.01 ± 3.81 ^a^	11.67 ± 5.89 ^b^	0.15 ± 0.01 ^d^	0.12 ± 0.00 ^d^
*Torulaspora delbrueckii* Disva 254	ND	ND	0.22 ± 0.02 ^c^	32.95 ± 3.54 ^a^	13.33 ± 1.18 ^b^	0.20 ± 0.01 ^b^	0.49 ± 0.00 ^b^
*Lachacea thermotolerans* Disva 322	ND	ND	1.19 ± 0.00 ^b^	27.76 ± 0.36 ^b^	13.33 ± 3.54 ^b^	0.32 ± 0.03 ^a^	0.47 ± 0.02 ^b^
*Saccharomyces cerevisiae* US-05	ND	ND	0.08 ± 0.03 ^d^	25.71 ± 0.18 ^b^	28.33 ± 5.89 ^a^	0.20 ± 0.00 ^b^	0.54 ± 0.01 ^a^

* ND—non-detectable.

**Table 3:** The main analytical characteristics of products from BSG + GJ wort. Data are the means ± SD. Data with different superscript letters (^a,b,c,d,e^) within each column (Duncan tests; *p* < 0.05). The initial BSG + GJ wort characteristics were glucose (16.73 g/L); sucrose (0.46 g/L); maltose (3.91 g/L); YAN (53.91 mg/L); polyphenols (75.83 mg/L).

Fermentations Trials	Glucose (g/L)	Sucrose (g/L)	Maltose (g/L)	YAN (mg/L)	Polyphenols (mg/L)	Lactic Acid (g/L)	Ethanol (% *v*/*v*)
*Wickeramhomyces anomalus* Disva 2	ND *	ND	2.21 ± 0.04 ^a^	28.14 ± 0.18 ^b^	19.17 ± 7.07 ^b^	0.17 ± 0.00 ^b^	1.32 ± 0.01 ^c^
*Pichia kluyveri* Disva 1076	1.91 ± 0.02 ^a^	0.20 ± 0.00 ^a^	0.90 ± 0.03 ^bc^	40.51 ± 0.82 ^a^	50.83 ± 2.36 ^a^	0.18 ± 0.01 ^b^	0.82 ± 0.01 ^e^
*Torulaspora delbrueckii* Disva 254	0.01 ± 0.00 ^b^	ND	0.84 ± 0.00 ^c^	15.90 ± 0.63 ^e^	1.67 ± 1.18 ^d^	0.18 ± 0.00 ^b^	1.66 ± 0.06 ^a^
*Lachancea thermotolerans* Disva 322	0.01 ± 0.00 ^b^	ND	0.95 ± 0.00 ^b^	25.19 ± 0.36 ^c^	6.67 ± 1.18 ^c^	0.85 ± 0.04 ^a^	1.40 ± 0.00 ^b^
*Saccharomyces cerevisiae* US-05	ND	ND	2.28 ± 0.02 ^a^	22.76 ± 1.27 ^d^	23.33 ± 1.18 ^b^	0.19 ± 0.00 ^b^	1.10 ± 0.00 ^d^

* ND—non-detectable.

**Table 4:** The main volatile compounds (mg/L) in beers coming from BSG wort. Data are means ± SD of three independent experiments. Data with different superscript letters (^a,b,c^) within each row (Duncan tests; *p* < 0.05). In parentheses: the odor active values (OAVs) of the compounds *.

Volatile Compounds mg/L	*Wickeramhomyces anomalus* Disva 2	*Pichia kluyveri* Disva 1076	*Torulaspora delbrueckii* Disva 254	*Lachancea thermotolerans* Disva 322	*Saccharomyces cerevisiae* US05
Ethyl butyrate (0.14–0.37) *	0.14 ± 0.01 ^c^	0.18 ± 0.07 ^c^	0.43 ± 0.06 ^b^	0.74 ± 0.10 ^a^	0.73 ± 0.07 ^a^
Isoamyl acetate (0.30–0.72) *	0.63 ± 0.01 ^b^	0.30 ± 0.03 ^c^	0.67 ± 0.05 ^b^	0.81 ± 0.09 ^a^	0.92 ± 0.03 ^a^
Ethyl hexanoate (0.17–0.20) *	0.03 ± 0.03 ^a^	0.04 ± 0.03 ^a^	0.05 ± 0.01 ^a^	0.03 ± 0.03 ^a^	0.03 ± 0.01 ^a^
Hexanol	ND **	ND	0.06 ± 0.01 ^a^	0.02 ± 0.00 ^b^	ND
Ethyl octanoate (0.2–0.9) *	0.01 ± 0.00 ^b^	ND	0.01 ± 0.00 ^b^	0.02 ± 0.00 ^a^	0.02 ± 0.00 ^a^
Linalool (0.0006–0.001) *	0.06 ± 0.04 ^a^	0.02 ± 0.01 ^a^	0.02 ± 0.00 ^a^	0.03 ± 0.03 ^a^	0.01 ± 0.00 ^a^
Diethylsuccinate (1.2) *	0.01 ± 0.00 ^b^	0.01 ± 0.00 ^b^	0.01 ± 0.00 ^b^	0.01 ± 0.00 ^b^	0.02 ± 0.01 ^a^
Phenyl ethyl acetate (3–5) *	0.39 ± 0.22 ^a^	0.77 ± 0.16 ^a^	0.44 ± 0.19 ^a^	0.41 ± 0.28 ^a^	0.54 ± 0.15 ^a^
Nerol (0.01) *	0.01 ± 0.00 ^a^	0.01 ± 0.00 ^a^	0.01 ± 0.00 ^a^	0.01 ± 0.00 ^a^	0.01 ± 0.00 ^a^
Geraniol (1.1) *	0.01 ± 0.00 ^b^	0.02 ± 0.01 ^b^	0.02 ± 0.00 ^b^	0.01 ± 0.01 ^b^	0.05 ± 0.00 ^a^
β-phenyl ethanol (1.0–1.88) *	0.48 ± 0.06 ^b^	0.18 ± 0.06 ^c^	0.39 ± 0.05 ^bc^	0.76 ± 0.23 ^a^	0.52 ± 0.04 ^b^

** ND—non-detectable. Retention times of the compounds in Table S1 (Supplemental Materials).

**Table 5:** The main volatile compounds (mg/L) in fermentation trials using BSG + GJ wort. Data are means ± SD from three independent experiments. Data with different superscript letters (^a,b,c,d^) within each row (Duncan tests; *p* < 0.05). * In parentheses: the odor active values (OAVs) of the compounds.

Volatile Compounds mg/L	*Wickeramhomyces anomalus* Disva 2	*Pichia kluyveri* Disva 1076	*Torulaspora delbrueckii* Disva 254	*Lachancea thermotolerans* Disva 322	*Saccharomyces cerevisiae* US05
Ethyl butyrate (0.14–0.37) *	0.35 ± 0.00 ^cd^	0.23 ± 0.07 ^d^	0.46 ± 0.04 ^bc^	0.79 ± 0.06 ^a^	0.54 ± 0.12 ^b^
Isoamyl acetate (0.30–0.72) *	0.79 ± 0.07 ^c^	0.85 ± 0.03 ^bc^	0.93 ± 0.10 ^bc^	1.04 ± 0.07 ^ab^	1.21 ± 0.16 ^a^
Ethyl hexanoate (0.17–0.20) *	0.05 ± 0.00 ^d^	0.06 ± 0.01 ^d^	0.22 ± 0.02 ^b^	0.15 ± 0.01 ^c^	0.34 ± 0.02 ^a^
Hexanol	ND **	ND	0.24 ± 0.03 ^a^	0.22 ± 0.01 ^a^	0.01 ± 0.00 ^b^
Ethyl octanoate (0.2–0.9) *	ND	0.01 ± 0.00 ^b^	0.01 ± 0.00 ^b^	0.02 ± 0.00 ^a^	0.02 ± 0.00 ^a^
Linalool (0.0006–0.001) *	0.03 ± 0.03 ^a^	0.02 ± 0.02 ^a^	0.01 ± 0.00 ^a^	0.02 ± 0.00 ^a^	0.04 ± 0.01 ^a^
Diethylsuccinate (1.2) *	0.01 ± 0.00 ^b^	0.02 ± 0.01 ^b^	0.06 ± 0.00 ^a^	0.01 ± 0.00 ^b^	0.04 ± 0.00 ^b^
Phenyl ethyl acetate (3–5) *	0.47 ± 0.05 ^b^	0.02 ± 0.01 ^c^	1.33 ± 0.50 ^a^	0.37 ± 0.05 ^b^	0.06 ± 0.05 ^c^
Nerol (0.01) *	ND	0.01 ± 0.00 ^a^	0.01 ± 0.00 ^a^	ND	0.01 ± 0.00 ^a^
Geraniol (1.1) *	0.01 ± 0.00 ^a^	0.02 ± 0.00 ^a^	0.02 ± 0.00 ^a^	0.05 ± 0.00 ^a^	0.10 ± 0.09 ^a^
β-phenyl ethanol (1.0–1.88) *	1.02 ± 0.28 ^a^	0.40 ± 0.10 ^b^	1.14 ± 0.23 ^a^	0.68 ± 0.20 ^ab^	0.42 ± 0.10 ^b^

** ND—non-detectable.

## Data Availability

Data are contained within the article or Supplementary Material.

## Supplementary Materials

The following supporting information can be downloaded at: https://www.mdpi.com/article/10.3390/foods13040505/s1, Table S1: Retention time of the volatile compounds evaluated.
