# Supplementary material for: Recycled Brewer’s Spent Grain (BSG) and Grape Juice: A New Tool for Non-Alcoholic (NAB) or Low-Alcoholic (LAB) Craft Beer Using Non-Conventional Yeasts
Source: Foods. 2024 Feb 6;13(4):505. doi: 10.3390/foods13040505 (PMC10887606; doi:10.3390/foods13040505)
Supplement: Supplementary file 1 [file foods-13-00505-s001.zip › foods-2802457-supplementary.pdf]

Table S1. Retention time of the volatile compounds evaluated

Volatile compounds mg/L	(Retation Time min)
Ethyl butyrate (0.14–0.37) *	7.151
Isoamyl acetate (0.30–0.72) *	9.082
Ethyl hexanoate (0.17–0.20) *	15.219
Hexanol	21.807
Ethyl octanoate (0.2–0.9) *	25.456
Linalool (0.0006–0.001) *	30.970
Diethylsuccinate (1.2) *	32.933
Phenyl ethyl acetate (3–5) *	40.615
Nerol (0.01) *	41.648
Geraniol (1.1) *	41.987
β-phenyl ethanol (1.0–1.88) *	44.175
